# Assessment of Polycyclic Aromatic Hydrocarbon Exposure in Trainee Firefighters Using PAH CALUX Bioassay

**DOI:** 10.3390/toxics12110825

**Published:** 2024-11-18

**Authors:** Johanna Grünfeld, Peter Møller, Ulla Vogel, Simon Pelle Jensen, Vivi Kofoed-Sørensen, Maria Helena Guerra Andersen

**Affiliations:** 1 The National Research Centre for the Working Environment, 2100 Copenhagen, Denmark; 2 Department of Public Health, Section of Environmental Health, University of Copenhagen, 1014 Copenhagen, Denmark

**Keywords:** bioassay, firefighter, biomonitoring, skin wipes, urine

## Abstract

This work investigated the application of a reporter gene bioassay in assessing polycyclic aromatic hydrocarbon (PAH) exposure in trainee firefighters. In the PAH CALUX bioassay, the PAH-induced activation of the aryl hydrocarbon receptor in a reporter cell line is recorded by increased luminescence. A repeated measurement study was performed, collecting urine and skin wipe samples at two baseline sessions (spring and autumn) and after three firefighting sessions: one with wood fuel, one with gas fuel, and one without fire. The bioassay response was expressed as benzo[a]pyrene equivalents, which was compared to levels of 16 EPA criteria PAHs in skin wipe samples and 8 hydroxylated PAHs (OH-PAHs) in urine samples quantified by chromatography–tandem mass spectrometry techniques. Benzo[a]pyrene equivalents and PAH levels in skin wipes indicated larger exposure to PAHs during the wood session compared to the other sessions. The urine bioassay showed non-significant effect sizes after all sessions, whereas the chemical analysis showed increased OH-PAH levels after the gas session. The non-significant changes observed for the session without fire suggest a negligible exposure from contaminated gear. In conclusion, the bioassay response for skin wipes shows that trainee firefighters were exposed to higher levels of potentially toxic PAHs during the wood fire training session.

## 1. Introduction

Occupational firefighting has been associated with adverse health effects such as increased risks of cancer, cardiovascular disease, and reduced fertility in males [1,2,3,4,5,6]. The International Agency for Research on Cancer (IARC) has classified occupational exposure as a firefighter as carcinogenic to humans (Group 1) [7].

Firefighters are exposed to a wide range of chemicals and particulate matter during training and firefighting activities. A central source of exposure is the smoke from fires and exhaust from combustion engines. The smoke contains a large number of toxic chemicals, including polycyclic aromatic hydrocarbons (PAHs) [7,8]. PAHs are a class of organic compounds commonly formed during the incomplete combustion of organic matter, and can exist both in gaseous form and adsorbed onto airborne particulates. The number of aromatic rings, their possible configurations, and their chemical modifications and derivatives make this group of chemicals highly numerous [9]. PAH properties have been shown to depend on the number of aromatic rings and functional groups, which also relate to their potential toxicity [9]. While most PAHs are categorized as ‘probable’ or ‘possible’ human carcinogens, some are considered to have sufficient evidence of causing cancer in humans [10], reinforcing the need to minimize firefighters’ exposure to PAHs wherever possible. Firefighters can be exposed to PAHs through inhalation, skin absorption, and ingestion. Also, during training, firefighters experience wood and gas fires that produce smoke with PAHs [11,12,13].

Firefighters’ exposure and the dominating route of exposure depend on several factors including (but not limited to) the type of firefighting activity, use of personal protective equipment (PPE), and physicochemical properties of the compounds in the smoke [14,15,16]. The quantity and components of the released combustion products depend on the chemical composition of the combusted fuel and the combustion completeness related to, for example, temperature and oxygen availability [17]. Even when firefighters use PPE, skin exposure of especially the face, neck, and wrists can occur by the penetration of contaminants through interface parts of the PPE, as well as by post fire contact with contaminated gear [15,18,19,20].

The exposure to PAHs by firefighters has typically been assessed by ambient air levels (person-borne sensors), PAHs in skin wipe samples, and biomarkers such as the urinary excretion of 1-hydroxypyrene [21,22,23]. However, these exposure markers do not adequately reflect the personal exposure to hazardous PAHs or the total burden of PAH exposure. PAHs comprise numerous compounds with diverse toxicological potentials [24]. Quantifications of PAHs and their metabolites and derivatives have generally been limited to chemical-specific analytical methods [25,26]. Analyzing only a subset of PAHs can cause uncertainties in the assessment of the overall exposure and potential toxicity of PAHs [20,27]. An improved estimation of the actual exposure and toxicity of PAHs can assist and simplify the work towards a safer working environment for firefighters.

Approaches using markers of effect may reduce the underestimation of the effects of PAH exposure. One example is the PAH CALUX bioassay, which quantifies the overall aryl hydrocarbon receptor (AhR)-induced biological response to PAH-related compounds [27]. The PAH CALUX bioassay utilizes a rat hepatoma (H4llE) cell line that is stably transfected with a luciferase reporter gene construct. The luciferase gene is controlled by a promoter from the mouse *Cyp1a1* gene containing the recognition motif of the AhR activation (AhR/ARNT complex), referred to as the dioxin- or AhR-responsive element (DRE or AHRE) [27,28,29]. Following binding of ligands such as PAHs, the AhR forms a heterodimeric transcription factor with the aryl hydrocarbon receptor nuclear translocator (ARNT) [30]. This ligand-activated transcription factor has a significant role in the metabolism of PAHs, e.g., as the major regulator of CYP1 expression, as well as mediating epigenetic effects related to tumor promotion and/or progression [31,32,33,34]. AhR activation plays a critical role in toxicity related to cancer [35], cardiovascular disease [36], and male infertility [37].

This work investigated the application of the PAH CALUX bioassay in assessing PAH exposure and related toxicity in trainee firefighters. A repeated measurement study was performed, collecting urine and skin wipe samples after three firefighting sessions: one without fire, and two with fire, using wood or gas fuels, respectively.

## 2. Materials and Methods

### 2.1. Subjects and Study Design

Seventeen healthy conscripts from a firefighting training program under the Danish Emergency Management Agency were enrolled in the study in 2023. The education proceeded for nine months, and the trainee firefighters were followed during baseline and three firefighting classes (“sessions”). The trainee firefighters comprised both men (*n* = 12) and women (*n* = 5) of at least 18 years of age (20.8 ± 1.5 years; average ± standard deviation) and were voluntarily recruited to participate in our study. Exclusion criteria were current smoking, pregnancy, underweight or obesity (18.5 ≤ BMI < 30), use of prescription medicines, and clinically diagnosed alcohol and drug abuse. The characteristics of the study subjects are presented in Supplementary Materials (Table S5).

The firefighting trial was designed as a pre–post crossover study, where the trainee firefighters were their own controls. The subjects were studied at two baseline time points and during three exposure scenarios: firefighting without fire, firefighting under wood fire, and firefighting under gas fire. The different fire conditions are used in current firefighting training programs in Denmark. Baseline samples were collected in the morning when the subjects were in classroom and not performing firefighting activities. Firefighting without fire involved exercises equivalent to firefighting training, including full equipment, in an environment without fire. In the wood fire session, standard European wooden pallet fuel was used and in the gas fire session, liquid petroleum gas (Kosan gas A/S) was used as fuel. The PPE was cleaned after each firefighting session. One of the baseline sessions took place during spring and the other during early autumn. The wood fire session took place in the spring and was thus compared to the spring baseline. The gas fire session took place in the autumn, and thus compared with the autumn baseline. The session without fire was randomized with some subjects having it in the spring (and compared to the spring baseline) and others having it in the autumn (and were compared to the autumn baseline).

### 2.2. Sample Collection

#### 2.2.1. Skin Wipe Samples

Skin wiping of the neck was performed to estimate the PAH deposition on the skin. Skin wiping was performed according to a methodology previously used [21] where the skin on the back of the neck was wiped with an “Alkoholswab” (70% isopropanol/water, Mediq Danmark A/S). Using a card template, a skin area of approximately 3 cm × 6 cm was wiped twice with the same wipe, once using each side of the wipe. The wiping was performed on the left and right side of the neck separately, resulting in two wipes per subject, pooled in extraction. However, during the gas fire session, only one wipe was sampled, which was corrected for in the subsequent analysis. Sampling was performed at the training facility by technical staff from the National Research Centre for the Working Environment, soon after completed firefighting sessions. Baseline samples were collected in the mornings before the sessions. Blank field wipe samples consisted of only one wipe, which was not used for wiping. The wipes were placed in 15 mL screw cap glass vials with foil-lined lids. Wipes were kept in the dark and stored at −18 °C until extractions.

#### 2.2.2. Urine Samples

Urine samples were collected to assess the internal PAH dose (resulting from combined inhalation, ingestion, and skin exposure). In order to minimize oral PAH intake, the subjects were asked not to eat grilled or smoked food within three days before the trial sessions. The urine sampled for the PAH CALUX bioassay and chemical analysis was collected in the morning the day after each firefighting session (without fire, wood fire, and gas fire), 13–16 h after the firefighting session was completed. It was the first urine in the morning in order to analyze the accumulated urinary hydroxylated PAH (OH-PAHs) excretion during the night. Baseline samples were collected in the same way as for the training sessions. The urine was sampled by the subjects themselves and delivered at the training facility. Urine samples were stored at −40 °C until extraction.

### 2.3. Sample Extraction

Skin wipe and urine samples were extracted prior to the analysis.

#### 2.3.1. Skin Wipe Samples

For the bioassay, the extraction of PAHs in skin wipes was performed by ultrasound-assisted extraction. Cyclohexane was added at a volume of 5 mL per wipe, resulting in 10 mL of cyclohexane per sample containing two wipes. The sample–solvent mixtures were placed in an ultrasonic bath for 60 min, and 3 mL of the single wipe samples (5 mL) and 6 mL of the double wipe samples (10 mL) were transferred to a new pre-weighted glass vial with a lid. DMSO was added to each sample at a volume of 200 μL. Evaporation of the samples were performed in a rotary evaporator called a “Rocket Evaporator”, by placing samples under vacuum while spinning at high speed. Samples were evaporated twice to remove all the cyclohexane. Samples were weighed before and after evaporation. Method blanks (single wipes) were treated the same way as skin wipe samples, including the ultrasonic bath and rotary evaporation. Extracts were stored at −20 °C until the analysis. For the chemical analysis, 100 µL internal stand (IS) deuterated PAH (PAH-Mix 9 deuterated, 100 µg/mL, in cyclohexane from LGC) was mixed with 900 µL from the 5 mL (one wipe sample) or 10 mL (two wipe sample) sample extract in cyclohexane.

#### 2.3.2. Urine Samples

Urine samples were thawed and homogenized prior to extraction for the bioassay. Samples from the different sessions and subjects were extracted in a randomized order. Aliquots of 10 mL from each sample were pipetted into vials. As PAHs are commonly excreted in a conjugated form [38], in order to enhance extraction efficiency, enzymatic deconjugation was performed [39]. Deconjugation was performed by adding 1 mL of 2.55 M sodium acetate buffer (pH 5) and 25 μL of β-glucuronidase from *Helix pomatia* to the urine and incubating overnight at 37 °C. Solid-phase extraction (SPE) was carried out using Waters HLB cartridges (6 mL) with 200 mg of a polymeric sorbent. The sorbents were conditioned with 5 mL of HPLC-grade Methanol (MeOH) followed by 5 mL ultrapure water. Samples were loaded into the cartridges and washed with 5 mL ultrapure water. Residual water was removed by blowing cartridges with nitrogen. Elution was performed with 2 × 5 mL of MeOH followed by 5 mL of a solution with 10% MeOH in Methyl tert-butyl ether (MTBE). Dimethyl Sulfoxide (DMSO) was added as a keeper solvent at a volume of 85 μL. Nitrogen was used to evaporate MeOH and MTBE. The eluent was brought up to 100 μL of DMSO by weight. The extracts were kept at −20 °C until the analysis. Method blanks of ultrapure water were treated the same way as urine samples, including deconjugation, and finally pooled into one vial.

### 2.4. PAH CALUX Bioassay

The PAH CALUX bioassay was performed according to the instructions from the manufacturer, BioDetection Systems (BDS, Amsterdam, The Netherlands), using both urine and skin wipe extracts as exposure. Prior to the analysis of samples from trainee firefighters, we performed validation experiments to ensure the implementation of reliable measurement (see Section 1 in Supplementary Materials for details). The cell line was cultured in α-MEM medium supplemented with 10% Fetal Bovine Serum (FBS) and 0.2% Pen-strep. The cell line was subcultured every Monday and Thursday. Three passages were completed before exposing the cells to extracts. Cells were seeded in 96-well cell culture plates in 100 μL of growth medium per well. The cells were seeded at a cell density of 60,000 cells per well (600,000 cells/mL). The seeded cells were incubated at 37 °C for 18–24 h before exposure.

The extracts and the standard solutions were thawed in a fridge overnight before exposure. The sample and reference dose medium were prepared on the day of exposure, at a volume of 100 μL. The samples, dissolved in DMSO, were added to the growth medium at a maximum level of 0.8%. However, dilution tests were performed to find suitable sample dilutions in order to match the standard curve. For urine samples, a dilution factor of 80 (0.01% DMSO) was mostly used, whereas a dilution factor of 70 was used for a few samples. For skin wipe samples, a dilution factor of 10 (0.08% DMSO) was chosen.

Method blanks were prepared using the same dilution factors as the samples of the plate. On each plate, a standard curve of benzo[a]pyrene (B[a]P) was made by adding a stock solution series in DMSO (prepared by the bioassay manufacturer) to the growth medium at a concentration of 0.8%. The sample and reference dose medium as well as the method blank were added to each plate in triplicates. All samples from the same subject were analyzed on the same plate. After the 4 h exposure, the medium was removed, and cells were washed with 150 μL PBS and lysed in 30 μL lysis reagent containing Triton-X100. The luciferase activity was measured as relative lights units (RLU) per well using the luminescence mode in a SpectraMax iD3 microplate reader (Molecular Devices LCC, San Jose, CA, USA). Of the urine samples, 75% were analyzed a second time. None of the skin samples were analyzed a second time.

Standard curves were created by fitting log-logistic regression models to RLU data across the standard concentration series. Standard curves were fitted to a four-parameter curve operated through a ready-to-use data treatment document (Excel) received by the bioassay manufacturer (see example in Figure S1 in Supplementary Materials). The fitting parameters of the curve were the upper limit; bottom; *EC*_50_, which is the concentration that induces a response corresponding to 50% of the maximum RLU (luciferase activity); and slope around the *EC*_50_ (see *EC*_50_ values in Table S1 in Supplementary Materials). The B[a]P equivalent for each sample was found by extrapolating the sample RLU to the standard curve while correcting for the method blank, using the equation provided by the manufacturer data treatment file:

(1) $$x_{1}={\left[\;\frac{(RLU_{S}-c_{SC})\;}{\left(d_{SC}-RLU_{S}\right)}\;\right]}^{1/b_{sc}}$$

In the equation, $x_{1}$ indicates the sample concentration of B[a]P and $RLU_{S}\;$ is the *RLU* of the sample. The factor $c_{SC}$ is the bottom parameter, $d_{SC}$ is the max (*d*), and $b_{sc}$ is the slope (*b*) of the standard curve (*sc*). The bottom parameter is a correction considering the method blank.

To allow for comparison between sample concentrations fitted through different standard curves (different plates), the B[a]P equivalents were adjusted for the dilution factor, and for the skin wipes, also the initial DMSO volumes.

### 2.5. Chemical Analysis

#### 2.5.1. Skin Wipe Samples

Sixteen PAHs in skin wipes were measured by gas chromatography–tandem mass spectrometry (GC-MS/MS); see the list and validation data in Supplementary Materials (Table S3).

The skin wipes were analyzed using a similar method as described in [21]. Shortly, extracts were analyzed by GC-MS/MS using a Bruker SCION triple quadrupole (TQ) (Bruker Daltonics, Bremen, Germany). A Bruker CP-8400 autosampler was used to add 1 μL of the sample extract to a programmed temperature vaporizing (PTV) injector at 280 °C, in the splitless mode, into the column with a He flow of 1 mL/min. The column was 60 m × 0.32 mm with a film thickness of 0.25 µm (HT8, SGE, Trajan Ltd., Victoria, Australia). The oven program of the GC was set at 80 °C for 1 min, increased to 280 °C with 10 °C/min (hold 20 min), and then increased to 310 °C with 20 °C/min (hold for 15 min). Complete separation was not possible for benzo[b]fluoranthene and benzo[k]fluoranthene, and by assuming equal response factors, they were calculated as the sum of both (Benzo[k+b]fluoranthene).

#### 2.5.2. Urine Samples

Measurements of eight OH-PAHs in urine were performed by online SPE–liquid chromatography–tandem mass spectrometry (SPE-LC-MS/MS); please see the list and validation data in Supplementary Materials (Table S4).

The chemical analysis of OH-PAHs urine was performed as described by Frederiksen et al. [40], with minor adjustments. Adjustments include a new LC-MS system and added SPE column backflush at the end of each analysis to improve column lifetime. Briefly, samples were thawed and homogenized, 800 µL was transferred to centrifuge tubes, 100 µL β-glucuronidase/buffer solution and 100 µL C13-labeled OH-PAH internal standard were added, followed by incubation overnight. Samples were then centrifuged and the supernatant was transferred to LC vials followed by the analysis by SPE-LC-MS/MS, using an Agilent 1290 infinity LC system and 6495C triple quadrupole MS. The column used for SPE was an Agilent Zorbax SB-C18 (2.1 × 30 mm, 3.5 μm) reversed-phase column. The injection volume was 100 μL and the analytic column a Phenomenex Synergi Hydro RP C18 (2.5 μm, 100 Å, 100 × 2 mm). Complete separation was not possible for 2- and 3-hydroxyphenantrene, and therefore they were reported as the sum of both.

Urine density was used to adjust for the subjects’ hydration levels. Urine density was determined by weighing and adjustments performed as described in [41]. Adjustment for creatinine concentration in urine was also performed and data are presented in Supplementary Materials (Figure S7 and Table S11).

### 2.6. Statistical Analysis

Data analyses of urine samples were performed for both density-adjusted and unadjusted urine data.

B[a]P equivalents < limit of detection (LOD) were set to zero. B[a]P equivalents < limit of quantification (LOQ) were imputed using the estimated concentration (“best estimate”), disregarding the LOQ in order to avoid many zeros or artificially higher levels. For urine samples that were analyzed a second time in the bioassay, the average of the B[a]P equivalents from the first and second measurement were applied in the data analysis.

To analyze the data and variable contributions, a linear mixed effects (LMEs) model was used through the R package lme4 [42]. The predictor (firefighting session) was nested in the ID, which refers to subjects. The model was applied for four outcome variables (skin wipe bioassay, skin wipe GC-MS/MS, urine bioassay, and urine SPE-LC-MS/MS). Investigating normality assumptions revealed a non-normal distribution of residuals. The response variables were therefore log- or cubic root-transformed, which reduced the skewness. Effect sizes between baseline and post-session PAH levels were calculated as percentage change based on the LME model estimates. Odds ratios for skin wipe results were obtained through logistic regression where standard scores (Z-scores) are the predictor and firefighting session the outcome (wood and gas = effect, baseline, and without fire = no effect). Z-scores were derived from bioassay results, GC-MS/MS results, and bioassay and GC-MS/MS results combined (mean).

## 3. Results

### 3.1. Levels of PAHs and B[a]P Equivalents After vs. Before Firefighting Sessions with Combustion of Wood or Gas, or Without Fire

#### 3.1.1. Skin Wipes

**Figure 1:**
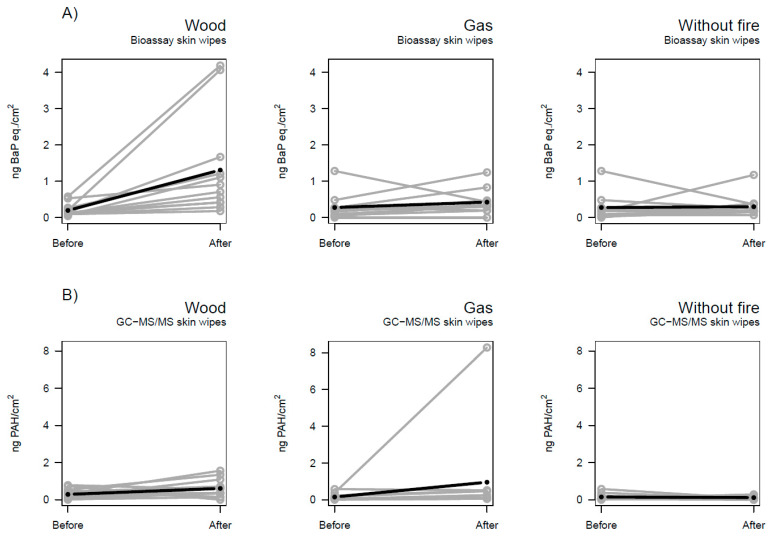
Levels of (**A**) B[a]P equivalents and (**B**) Ʃ16 PAHs (ng/cm^2^) for skin wipes before and after the three firefighting sessions (wood, gas, and without fire). Bold lines indicate the mixed effect linear regression. Samples were collected from repeated measurements where each point represents one measurement. Subjects (*n*) = 14 (wood) and 11 (gas, without fire).

Figure 1 depicts the changes in B[a]P equivalents (Figure 1A) and PAHs (Figure 1B) in skin wipe samples after each session. Variation between subjects can be observed for all three sessions (wood, gas, and without fire), to varying extents, especially for the bioassay results (Figure 1A). Some variation was caused by putative outliers, e.g., in the wood session for bioassay results (Figure 1A) and in the gas session for GC-MS/MS results (Figure 1B).

#### 3.1.2. Urine

**Figure 2:**
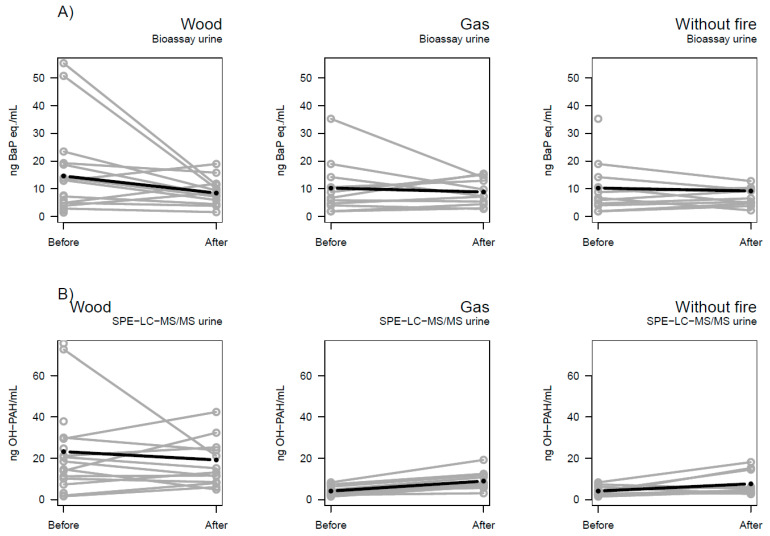
Levels of (**A**) B[a]P equivalents and (**B**) Ʃ8 OH-PAHs (ng/mL) for density-adjusted urine before and after the three firefighting sessions (wood, gas, and without fire). Bold lines indicate the mixed effect linear regression. Samples were collected from repeated measurements where each point represents one measurement. Subjects (*n*) = 14 (wood) and 11 (gas, without fire).

Figure 2 shows the changes in B[a]P equivalents (A) and OH-PAHs (B) in urine after each session compared to before. Individual variation is evident and for some subjects, an increase was observed. The variation was especially large at baseline (before session), and at least two putative outliers (>50 ng B[a]P eq./mL) were observed for the bioassay results (Figure 2A), and two (>70 ng OH-PAH/mL) among SPE-LC-MS/MS results (Figure 2B) (not corresponding to the same subjects for the two assays), for the wood session (spring baseline). Individual variation in the gas session is larger for the bioassay results (A) compared to SPE-LC-MS/MS results (B).

Adjustment for the self-reported consumption of grilled food and smoked food and exposure to smoke or second-hand smoke within the last three days did not change the outcome of the LME model (results are not shown).

Baseline levels can be found in the Supplementary Materials (Tables S6–S8).

### 3.2. Effect Sizes (Percentage Change) Between Baseline and After Firefighting Session

Percentage changes between baseline and post-session levels were calculated based on the LME model estimates. The aim was to quantify the difference visualized in Figure 1 and Figure 2. An extreme value was removed from the skin wipe GC-MS/MS dataset (gas session, value depicted in Figure 1B) to comply with the normality assumptions of the model (as the other transformation attempts, cubic root or logarithmic transformation, did not succeed in ensuring the normality of the residuals).

For skin wipe data in Table 1, significant changes (*p* < 0.05) were found for the wood session in both the bioassay data and GC-MS/MS data. In both cases, the measured PAHs and B[a]P eq. were increased post session as compared to baseline.

For urine samples, presented in Table 2, a significant increase in the session without fire did not result in significant changes for any of the analyses, neither for urine samples nor skin wipe samples.

### 3.3. Association Between PAH Level and Exposure Scenario

Logistic regression with Z-scores from PAH levels was used to obtain odds ratios, in order to enable comparisons across skin wipe datasets. Z-scores were calculated from exposure levels from three different skin wipe datasets (bioassay, GC-MS/MS, and bioassay and GC-MS/MS combined). Odds ratios are only presented for skin wipes (Table 3) as the LME model for urine largely results in non-significant effects.

All odds ratios and confidence intervals for skin wipes were statistically significant. The odds ratio was numerically higher for GC-MS/MS results than bioassay results. The highest odds ratio was seen for the combined bioassay and GC-MS/MS results. This indicates that the best prediction of exposure was obtained by using a combination of the bioassay and chemical analysis of PAHs.

For density-adjusted urine, the logistic regression analysis led to non-significant effects (OR < 1) (please see Table S12 in Supplementary Materials).

## 4. Discussion

In this study, we assessed PAH exposure of 17 volunteers during firefighter training in three different exposure scenarios. We evaluated PAH exposure by measuring dermal contamination through skin wipes and internal exposure via urinary excretion. PAH exposure was assessed using a bioassay quantifying B[a]P equivalents and by measurements of PAH content in skin wipes and PAH metabolites in urine using chromatography–tandem mass spectrometry. The study showed that the PAH CALUX bioassay may be considered reliable for assessment of PAH exposure on the skin in firefighting trials. The analysis of internal exposure via urine samples may have been affected by other factors, which we will discuss further.

The CALUX assay performance was benchmarked by the determination of EC_50_ for B[a]P. The mean EC_50_ derived from all B[a]P standard curve fits (2.35 ± 1.95 nM, Table S1 in Supplementary Materials) is comparable to the B[a]P EC_50_ of 4.56 ± 8.66 nM reported by [20] and that of 1.05 ± 0.31 nM [43] as well as the reported EC_50_ of 3 nM in [27].

### 4.1. Urine Samples and Uncertainties

For urine samples, the majority of the effect sizes in bioassay and chemical analysis (SPE-LC-MS/MS) data for the firefighting sessions were statistically non-significant, except for the effect found for the gas session in SPE-LC-MS/MS data (Table 2), with levels dominated by hydroxynaphthalenes (Table S10 in Supplementary Materials). The results indicate a substantial difference in the detection of firefighting exposure levels between the two analytical methods (bioassay and SPE-LC-MS/MS). In addition, a considerable interpersonal variation in exposure levels was observed (Figure 2). However, a large inter-personal variation in urinary 1-hydroxypyrene and other OH-PAHs between conscripts undergoing the same firefighting training program has also been reported in previous studies [21,40], suggesting that individual behavior during firefighting training is an important determinant of the PAH exposure.

For urine SPE-LC-MS/MS data (Figure 2B), baseline levels of OH-PAHs in the wood session were around four times higher than baseline for the gas session. In the urine bioassay data (Figure 2A), however, there was no substantial difference in baseline levels of B[a]P equivalents between the wood and the gas sessions. A high baseline with considerable variation between individuals, as in the SPE-LC-MS/MS data, complicates the interpretation of the data. The difference in baseline OH-PAH levels might be linked to seasonal variation in PAH exposure, as the wood session including baseline was performed in the spring whereas the gas session including baseline (and for the session without fire, for the majority of subjects) was performed in the autumn. The differences in baseline may also be related to ADME (Absorption, Distribution, Metabolization, and Excretion) processes, through either seasonal variation or any unknown factor. Possible short-term exposure can be air pollution, smoking, or dietary factors that the subjects did not report. Short-term factors that only affected baseline could have led to a false negative effect size for the wood session.

Creatinine concentration in urine was measured but did not provide better results for urine concentration adjustments (data can be found in Sections 7 and 8 in Supplementary Materials). Additionally, the high level of physical exercise during firefighting activities, which might have varied between sessions, could theoretically have affected creatinine excretion (see Figure S6 in Supplementary Materials).

### 4.2. Urinary Metabolite Excretion Rate

The successful investigation of the internal dose through urine metabolites sampling requires that the time of sampling is compatible with the excretion rates of the metabolites [44], also taking into account that urinary excretion rates comprise individual variation and uncertainties.

Urine samples in this study were collected 13–16 h after the end of the firefighting sessions. Urinary elimination half-lives of around 3–9 h have been reported for low-weight OH-PAHs such as naphthalene, fluorene, and phenanthrene [23,45,46]. Many previous studies that found significantly increased concentrations of 1-hydroxypyrene in urine after firefighting collected urine samples 3 to 12 h post fire [11,16,46,47]. Recently, we reported a peak excretion 4–6 h after the firefighting training session with other conscripts, which corresponds to 8–10 h after the first exposure [40]. Although the post-fire sampling time of this study might have been later as compared to the urinary half-lives and peaks of low-weight OH-PAHs, the SPE-LC-MS/MS data demonstrate the presence of low-weight OH-PAHs (including 1-hydroxynaphtalene and 2-hydroxynaphtalene) for the wood and gas sessions. Furthermore, the OH-PAHs were expected to accumulate during the night and therefore still be present in the first morning urine. However, the sampling time of this study might not have been optimal, possibly explaining the low levels of OH-PAHs after most training sessions. The sampling time may thus be a reason for the lack of effect of exposure seen in urinary data, including the bioassay.

### 4.3. Skin Wipe Samples

The results from the skin wipe samples are more predictable as compared to urine results. The bioassay and chemical analysis (GC-MS/MS) of PAHs in skin wipe samples from three different firefighter sessions suggested that the largest skin exposure of PAHs occurred during the wood session (Table 1). The exposure to PAHs by skin contamination in the gas session seemed to be low. Whether the PAH exposure on the skin is caused by penetration through the PPE, by direct deposition in the neck in between interface parts of the PPE, or by contact with contaminated gear [15,18,19] cannot be determined in this study. However, the low PAH levels and non-significant changes after the session without fire, during which the trainees also wore their PPE, suggest that the skin deposition mainly occurs during/after the fire sessions and not from pre-contaminated gear. The increase in exposure after the wood session was larger in the bioassay (>400%) compared to the GC-MS/MS (≈80%) results.

### 4.4. The Bioassay Response for Low- Versus High-Molecular-Weight PAHs

Different PAHs are expected to have different activity in the bioassay. As high-molecular-weight PAH metabolites are mainly excreted in feces [10,48], we expected more low-molecular-weight PAH metabolites in urine, such as hydroxynaphthalenes. However, low-molecular-weight PAHs are potentially weak ligands for the AhR [49] and might thus give a relatively low response in the bioassay as compared to higher-molecular-weight PAHs. This potential gap may be a part of the reason for the differences in the results for urine samples compared to skin wipe samples. A previous study by Beitel et al. [20], using the same PAH CALUX bioassay, also found significant increases in PAH levels in post-fire skin wipe samples (from neck and calf), whereas mostly non-significant changes were found for urine samples. Beitel et al. also investigated the bioassay response of fourteen OH-PAHs and six other related compounds (e.g., methoxyphenols) and only found a quantifiable agonistic response for seven OH-PAHs. The low-molecular-weight 2-hydroxynaphthalene, 2-hydroxyphenanthrene, and 4-hydroxyphenanthrene were among the seven OH-PAHs with a response in the bioassay [20], which somewhat contradicts the theory of low-molecular-weight metabolites being poor agonists in the bioassay. However, 1-hydroxynaphthalene and three of the hydroxy-phenanthrenes (also low-molecular-weight OH-PAHs) did not induce a response in the bioassay in [20]. Furthermore, the shown bioassay response for some of the low-molecular-weight OH-PAHs might still be lower in relation to their potential presence in the sample, in comparison to high-molecular-weight OH-PAHs. As also stated by Beitel et al. [20], more research is needed to fully understand the potency of specific PAHs, OH-PAHs, and other exogenous and endogenous compounds present in the complex urine matrix in the bioassay in order to help clarify applicability of the bioassay. Ultimately, the different responses reported by the bioassay for individual PAHs indicate the bioassay’s differential sensitivity to certain PAHs.

### 4.5. Correlation Between Bioassay and Chemical Analysis

The bioassay detects the PAHs that bind to the AhR whereas chemical analyses quantify selected PAHs in a mixture. Consequently, these analytical approaches have different scopes, revealing different aspects of the exposure. Because of this, and as the bioassay may possess varying potency for different PAHs and OH-PAHs, and potentially lower potency for the dominating OH-PAHs found through SPE-LC-MS/MS in urine, we would not expect a linear correlation between the data from bioassay and chemical analyses (GC-MS/MS and SPE-LC-MS/MS). The correlation between bioassay and chemical analysis data was found to be lower for urine results than skin wipe results, although the *r*-values were close to zero for both urine and skin wipe data (Table S12 in Supplementary Materials).

In contrast to the current study, Beitel et al. [20] found a statistically significant correlation (Spearman correlation coefficient, r = 0.64) between the bioassay response for urine samples and chemical analysis (GC-MS/MS) of 20 OH-PAHs. However, only 1% of the bioassay response was calculated (through predicted B[a]P equivalence) to be caused by the quantified OH-PAHs and thus 99% by other unknown AhR-stimulating compounds. This was not surprising according to Beitel et al. since OH-PAHs are linked to only one of the metabolic pathways and only a part of all existing OH-PAHs were quantified through the chemical analysis. Beitel et al. did not report on the correlation between the bioassay response of skin wipe samples and chemical analysis.

Odds ratios (Table 3) for skin wipe samples were calculated to compare the prediction of exposure between the bioassay and GC-MS/MS and datasets (adjusted to Z-scores and used as predictors), where the outcome was based on the expected exposure during the firefighting sessions (wood and gas session = exposure, baseline and without fire = no exposure). Between the bioassay and GC-MS/MS data, the highest odds ratio and thus best prediction of exposure were found for the GC-MS/MS data. However, odds ratios were also obtained for the combined datasets (mean), which gave an even higher odds ratio. Using a combination of the bioassay and chemical analysis is thus a better predictor of PAH exposure than either of the analysis/assay alone, which highlights the value of including different analyses. Nevertheless, to be able to draw any general conclusion on these predictions, they need to be tested on a new dataset or in a new study, preferably with more subjects.

### 4.6. Strengths and Limitations

The study focuses on a training setup, allowing for better control of the firefighting activities and the subjects, who are conscripts residing in the same facilities and therefore sharing many daily life factors. Additionally, the repeated measurements design provides a strong experimental design that controls for many sources of individual variation. Nevertheless, the timing and duration of the firefighting exercises may vary between subjects, which was not controlled. The variation in exposure levels and the small contrast between baseline and exposure, which for the urine samples might be affected by the sampling time, along with the small number of subjects, present limitations for our study that could particularly impact the results regarding urine samples.

## 5. Conclusions

B[a]P equivalents by the PAH CALUX bioassay and PAH levels by the chemical analysis in skin wipes from trainee firefighters indicated that the firefighting training session using wood fuel resulted in larger exposure to PAHs as compared to a firefighting session using gas fuel. Results from urinary samples from the same firefighting sessions were more unclear with mostly non-significant effects. The urine bioassay showed non-significant exposure levels after both the gas and wood sessions, whereas the chemical analysis showed increased OH-PAH levels only after the gas session. Small and non-significant changes were observed for a firefighting session without fire, suggesting only minor exposure from contaminated PPE. The results indicate that the PAH CALUX bioassay is useful for assessing the toxic potency of PAHs in skin wipe samples (OR = 8.5, 95% CI: 1.8, 39.4), especially in combination with the chemical analysis (OR = 59.6, 95% CI: 4.5, 786.1). For urine samples, further investigations are needed to clarify uncertainties related to the response. Especially human biomonitoring studies with a larger exposure gradient (e.g., real-life firefighting situations) and/or emissions containing a higher proportion of carcinogenic PAHs (i.e., high-molecular-weight PAHs with four or more aromatic rings) might achieve clearer results in the PAH CALUX assay. Ultimately, both the bioassay response and chemical analysis for skin wipes show that firefighter trainees were exposed to higher levels of potentially toxic PAHs during the firefighting session with wood fuel.

## Figures and Tables

**Table 1:** The percentage change and confidence intervals (CIs) per type of fire, indicating the difference in PAH content in skin wipes between before and after the three firefighting sessions. Calculations were based on the LME model output. Bold text indicates statistically significant values (*p* < 0.05).

Skin Wipe Analysis	Unit	Wood After vs. Before Session		Gas After vs. Before Session		Without Fire After vs. Before Session	
		% Change (95% CI)	*p*-Value	% Change (95% CI)	*p*-Value	% Change (95% CI)	*p*-Value
Skin wipe bioassay	ng B[a]P eq./cm^2^	**412.1 (181.8; 747.6)**	**3.0 × 10^−6^**	53.9 (−20.6; 189.1)	0.21	29.1 (−32.4; 146.6)	0.45
Skin wipe GC-MS/MS	ng PAH /cm^2^	**82.9 (0.3; 212.1)**	**0.05**	54.8 (−31.0; 231.0)	0.30	−15.9 (−55.0; 86.3)	0.67

**Table 2:** The percentage change and confidence intervals (CIs) per type of fire, indicating the difference in PAH metabolites for density-adjusted urine between before and after the three firefighting sessions. Calculations were based on the LME model output. Bold text indicates statistically significant values (*p* < 0.05).

Urine Analysis	Unit	Wood After vs. Before Session		Gas After vs. Before Session		Without Fire After vs. Before Session	
		% Change (95% CI)	*p*-Value	% Change (95% CI)	*p*-Value	% Change (95% CI)	*p*-Value
Urine Bioassay	ng B[a]P eq./mL	−25.2 (−53.0; 19.2)	0.22	5.6 (−37.4; 78.4)	0.84	−8.2 (−46.5; 57.4)	0.76
Urine SPE-LC-MS/MS	ng OH-PAH/mL	2.8 (−34.1; 60.4)	0.90	**123.9 (35.9; 268.9)**	**0.002**	56.8 (−6.2; 162.2)	0.086

**Table 3:** Odds ratios for skin wipe results obtained by logistic regression. Odds ratios were calculated using standard scores (Z-scores) as predictors and firefighting session as outcome (wood and gas = 1/effect, baseline and without fire = 0/no effect). Z-scores were derived from bioassay results, GC-MS/MS results, and bioassay and GC-MS/MS results combined (mean), and analyzed as continuous data.

Skin Wipes	Odds Ratio	95% CI
Skin Wipes Bioassay	8.5	1.8; 39.4
Skin Wipes GC-MS/MS	25.9	2.3; 288.1
Skin Wipes Combined (Bioassay and GC-MS/MS)	59.6	4.5; 786.1

## Data Availability

The data presented in this study are available on request from the corresponding author due to privacy/ethical restrictions.

## Supplementary Materials

The following supporting information can be downloaded at: https://www.mdpi.com/article/10.3390/toxics12110825/s1, Section 1: Bioassay implementation (Figures S1–S4 and Tables S1 and S2). Section 2: List of PAHs measured in skin wipes by GC-MS/MS and validation data (Table S3). Section 3: List of OH-PAHs measured in urine by SPE-LC-MS/MS and validation data (Table S4). Section 4: Characteristics of study subjects (Table S5). Section 5: Baseline levels (Tables S6-S8). Section 6: Levels per session for individual PAH and OH-PAH (Tables S9 and S10). Section 7: Density-adjustment justification (Figures S5 and S6). Section 8: Levels of PAHs and B[a]P equivalents for creatinine-adjusted urine (Figure S7). Section 9: Effect sizes between baseline and after firefighting session for creatinine-adjusted urine (Table S11). Section 10: Odds ratios for urine (Table S12). Section 11: Correlation bioassay and chemical analysis (Figures S8 and S9).
