# Supplementary material for: Assessment of Polycyclic Aromatic Hydrocarbon Exposure in Trainee Firefighters Using PAH CALUX Bioassay
Source: Toxics. 2024 Nov 18;12(11):825. doi: 10.3390/toxics12110825 (PMC11598809; doi:10.3390/toxics12110825)
Supplement: Supplementary file 1 [file toxics-12-00825-s001.zip › toxics-3253213-supplementary.pdf]

Supplementary material to:

Assessment of Polycyclic Aromatic Hydrocarbon Exposure in Trainee Firefighters using PAH CALUX Bioassay

Johanna Grünfeld [1, 2], Peter Møller [2], Ulla Vogel [1], Simon Pelle Jensen [1], Vivi Kofoed-Sørensen [1] and Maria Helena Guerra Andersen [1, *]

[1] The National Research Centre for the Working Environment, Copenhagen, Denmark

[2] University of Copenhagen, Department of Public Health, Section of Environmental Health, Copenhagen, Denmark

*Corresponding author: mga@nfa.dk (Maria Helena Guerra Andersen).

Contents

1. Bioassay implementation 2
   1.1 B[a]P standard curves 2
   1.2 Dilution curves 3
   1.3 B[a]P-spiked urine samples 4
   1.4 Bioassay repeatability 4
2. List of PAHs measured in skin wipes by GC-MS/MS and validation data 5
3. List of OH-PAHs measured in urine by SPE-LC-MS/MS and validation data 6
4. Characteristics of study subjects 6
5. Baseline levels 7
6. Levels per session for individual PAH and OH-PAH 8
7. Density-adjustment justification 10
8. Levels of PAHs and B[a]P equivalents for creatinine-adjusted urine 11
9. Effect sizes between baseline and after firefighting session for creatinine-adjusted urine 12
10. Odds ratios for urine 12
11. Correlation bioassay and chemical analysis 12

References 13

1. Bioassay implementation

Initial experiments were performed to gain some insight on the robustness of the bioassay. In addition to analyzing standard curves of B[a]P, the bioassay response was examined in two other ways; by (1) dilution curves for urine and skin samples and by (2) spiking pooled urine with different concentrations of the standard stock solutions. Each of the experiments were only performed once.

1.1 B[a]P standard curves

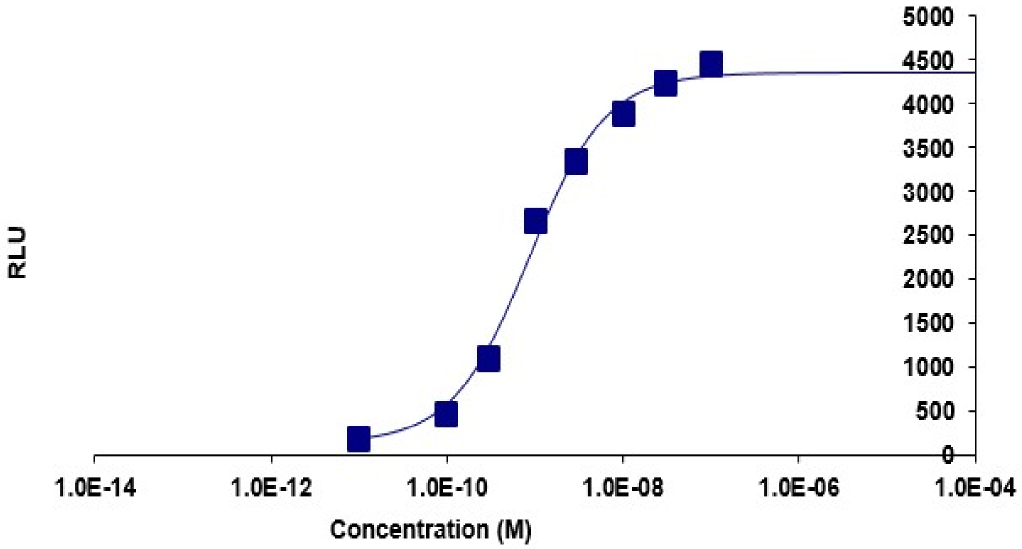

Figure S1. Luciferase activity in RLU as a function of exposure to B[a]P standard concentrations (M) in one of the PAH CALUX bioassay experiments. The standard curve was used to analyze urine samples from firefighters and obtained by fitting a four-parameter log-logistic model using the bioassay manufacturer's excel data treatment file. Each symbol represents one measurement. EC_{50} = 7.76 x 10^{-10} and r^2 = 0.997.

All standard curves were fitted with a sigmoidal shape, demonstrating an increased response in luciferase activity as relative lights units (RLU)) with increasing concentration of B[a]P.

Table S1. Mean EC50 values derived from the sigmoidal fit equation of all 17 standard curves (skin + urine) and the mean EC50 values for the 6 skin wipe samples and 11 urine sample plates separately.

Mean EC50 (M) **- Skin + urine**	S.D **- Skin + urine**	Mean EC50 (M) **- Skin**	S.D **- Skin**	Mean EC50 (M) **- Urine**	S.D **- Urine**
2.35 x 10^{-9}	1.95 x 10^{-9}	2.30 x 10^{-9}	8.07 x 10^{-10}	2.38 x 10^{-9}	2.35 x 10^{-9}

1.2 Dilution curves

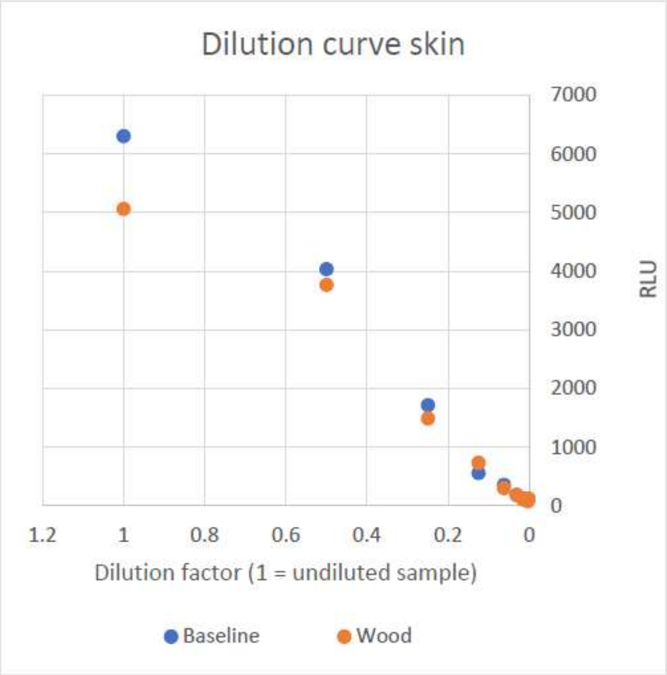

Figure S2. Luciferase activity in RLU as a function of increasing dilution factor of skin wipe samples in the PAH CALUX bioassay. 1 = undiluted sample. Samples from the baseline and wood session from the same subject was used for dilution curves. Dilutions were increasing with 2-fold steps. Each symbol represent one measurement.

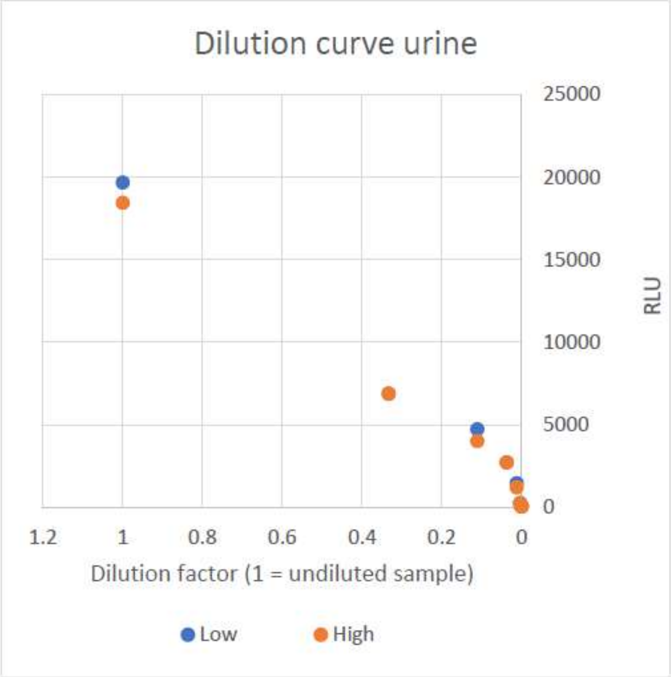

Figure S3. Luciferase activity in RLU as a function of increasing dilution factor of urine samples in the PAH CALUX bioassay. 1 = undiluted sample. Pooled urine from previous conscripts denoted "Low" or "High" based on their 1-OH-PYR concentrations were used for dilution curves. Dilutions were increasing with 3-fold steps. Each symbol represent one measurement.

The dilution curves for urine and skin wipe samples show decreasing RLU with increasing dilution factor, indicating a dose-response relationship also for biological samples.

1.3 B[a]P-spiked urine samples

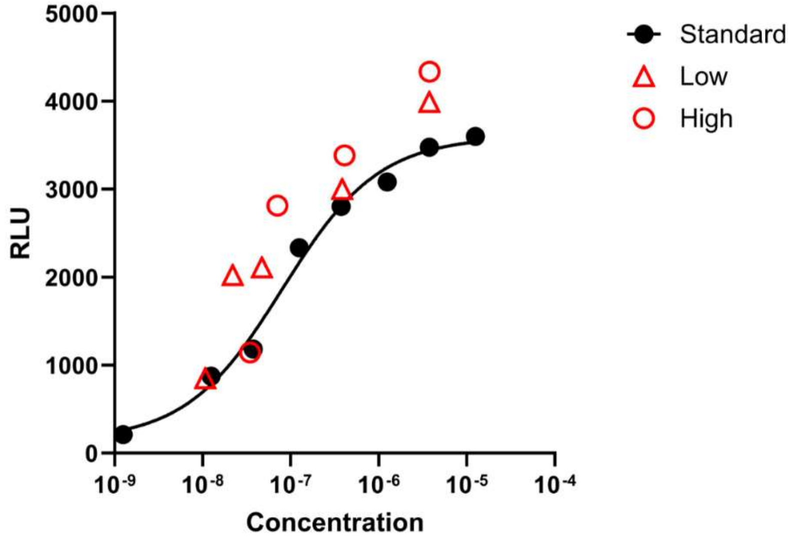

Figure S4. Luciferase activity in RLU in pooled urine spiked with different B[a]P standard concentrations, analysed with the PAH CALUX bioassay. The urine used for spiking was pooled into two groups, "high" and "low" based on the 1-OH-PYR concentration. A log-logistic non-linear regression was performed in GraphPad to obtain the sigmoidal standard curve.

The urine spiked with B[a]P, for which the "low" and "high" represent two pooled urine samples based on the concentration of 1-hydroxypyrene, indicates a higher response for spiked urine compared to non-spiked urine.

1.4 Bioassay repeatability

Repeatability for the bioassay was calculated in percent using the equation:

$$Standard\ deviation\ x\ 100/mean$$

Three different repeatability measures were calculated based on the following sample types:

- **Control**: Pooled urine from conscripts in a previous campaign of the same education program. The urine sample was analysed in the bioassay in a high concentration (dilution factor of 50 compared to a dilution factor of 70/80 for other urine samples). The reapeatability was calculated from 5 measurements.
- **Lowest standard concentration**: Lowest concentration of B[a]P among a series of eight concentrations, mixed in DMSO. The reapeatability was calculated from 17 measurements.
- **Highest standard concentration**: Highest concentration of B[a]P among a series of eight concentrations, mixed in DMSO. The reapeatability was calculated from 17 measurements.

Table S2. Bioassay repeatability (%) calculated from standard deviation x 100/mean. The repeatability was calculated from three different sample types; urine control (n=5), lowest standard concentration (n=17) and highest standard concentration (n=17).

Repeatability - bioassay		
Control (high concentration of urine)	Lowest standard concentration	Highest standard concentration
44 %	38 %	78 %

2. List of PAHs measured in skin wipes by GC-MS/MS and validation data

Table S3. The applied identification ions and the estimated limits of detection and quantification of the individual PAHs

	**Monoisotopic mass**	**Quantification ion**	**LOD analytical**	**LOD Skin conc.**	**LOQ analytical**	**LOQ Skin conc.**
	Da	Da	ng/mL	ng/cm^2	ng/mL	ng/cm^2
**Naphthalene**	128.0626	128	0.3	0.1	1.1	0.3
**Acenaphthylene**	152.0626	152	0.7	0.2	2.4	0.7
**Acenaphthene**	154.0783	154	2.0	0.5	6.6	1.8
**Fluorene**	166.0783	166	0.2	0.0	0.6	0.2
**Phenanthrene**	178.0783	178	0.4	0.1	1.4	0.4
**Anthracene**	178.0783	178	0.4	0.1	1.2	0.3
**Fluoranthene**	202.0783	202	0.5	0.1	1.8	0.5
**Pyrene**	202.0783	202	0.5	0.2	1.8	0.5
**Benz(a)anthracene**	228.0939	228	0.6	0.2	1.9	0.5
**Chrysene**	228.0939	228	0.5	0.1	1.6	0.5
**Benzo(b + k)fluoranthene**	252.0939	252	1.7	0.5	5.7	1.6
**Benzo(a)pyrene**	252.0939	252	2.5	0.7	8.2	2.3
**Dibenz(a,h)anthracene**	278.1096	278	31.8	8.8	105.8	29.4
**Ideno(1,2,3-cd)pyrene**	276.0939	276	10.1	2.8	33.6	9.3
**Benzo(g,h,i)perylene**	276.0939	276	14.0	3.9	46.6	13.0

LOD, limit of detection; LOQ, limit of quantification

3. List of OH-PAHs measured in urine by SPE-LC-MS/MS and validation data

Table S4. Validation results for linear range, LOQ and accuracy of the individual OH-PAHs from our laboratory, as published in Frederiksen et al., 2024 [1] (the presented validation results are taken from the reference [1], where more data is available)

Metabolite	Linear range (ng/mL urine)	LOQ (ng/mL) [signal-to-noise]	Accuracy % (n=11)
**1-OH-NAP**	0.1-40	0.1 [S/N: 32]	103.4 ± 4.7
**2-OH-NAP**	0.1-40	0.1 [S/N: 154]	101.9 ± 2.4
**2-OH-FLU**	0.01-2.5	0.01 [S/N: 122]	104.3 ± 2.7 *
**2+3-OH-PHE**	0.02-5	0.02 [S/N: 177]	97.6 ± 2.8
**1-OH-PHE**	0.01-2.5	0.01 [S/N: 56]	104.8 ± 5.6
**4-OH-PHE**	0.01-2.5	0.01 [S/N: 63]	96.2 ± 6.4
**1-OH-PYR**	0.01-2.5	0.01 [S/N: 86]	91.6 ± 5.9

1-OH-NAP, 1-hydroxynaphthalene; 2-OH-NAP, 2-hydroxynaphthalene; 2-OH-FLU, 2-hydroxyfluorene; 2+3-OH-PHE, 2- and 3-hydroxyphenanthrene; 1-OH-PHE, 1-hydroxyphenanthrene; 4-OH-PHE, 4-hydroxyphenanthrene; 1-OH-PYR, 1-hydroxypyrene.

*2-OH-FLU in relation to the reference material certificate from 2015, as described in [1]

4. Characteristics of study subjects

Table S5. Characteristics of the study subjects at the first baseline. Values are median and IQR.

Variable	**Females** (n=5)	**Males** (n=12)	**Total** (n=17)
**Age (years)**	20 [20, 22]	21 [20, 21]	21 [20, 21]
**Height (cm)**	170 [167, 170]	181.5 [177.0, 190.2]	179 [172, 187]
**Weight (Kg)**	67 [67, 76]	80 [74.2, 88.5]	77 [71, 88]
**BMI (Kg/m^2)**	24.5 [24.0, 26.3]	24.4 [22.4, 26.4]	24.5 [23.2, 26.3]

BMI, body mass index; IQR, inter-quartile range (25th and 75th percentiles).

5. Baseline levels

Table S6. Baseline mean and 10th to 90th percentiles for skin wipe concentrations of B[a]P equivalents and Σ16 PAHs (ng/cm^2). Baseline was measured before the three firefighting sessions; wood, gas and without fire. Baseline levels was measured during spring and autumn, where the spring baseline was used for comparison to the wood fire session and the autumn baseline were used for comparison to the gas fire session and the session without fire.

**Skin analysis**	**Unit**	**Baseline Mean (10th to 90th percentiles)**	
		Wood baseline	Gas & Without fire baseline
Skin bioassay	ng B[a]P eq./cm^2	0.20 (0.07-0.45)	0.28 (0.03-0.48)
Skin GC-MS/MS	ng PAH/cm^2	0.29 (0.04-0.63)	0.15 (0.03-0.37)

B[a]P eq, Benzo[a]pyrene equivalents; GC-MS/MS, Gas chromatography – tandem mass spectrometry; PAH, polycyclic aromatic hydrocarbons

Table S7. Baseline mean and 10th to 90th percentiles for density-adjusted urine concentrations of B[a]P equivalents and Σ8 OH-PAHs (ng/mL). Baseline was measured before the three firefighting sessions; wood, gas and without fire. Baseline levels were measured during spring and autumn, where the spring baseline was used for comparison to the wood fire session and the autumn baseline was used for comparison to the gas fire session and the session without fire.

**Urine analysis** density-adjusted	**Unit**	**Baseline Mean (10th to 90th percentiles)**	
		Wood baseline	Gas & Without fire baseline
Urine bioassay	ng B[a]P eq./mL	14.61 (2.61-34.33)	10.26 (1.89-18.93)
Urine SPE-LC-MS/MS	ng OH-PAH/mL	23.20 (2.69-51.77)	3.71 (2.04-6.37)

B[a]P eq, Benzo[a]pyrene equivalents; SPE-LC-MS/MS, solid phase extraction- liquid chromatography – tandem mass spectrometry; OH-PAH, hydroxylated polycyclic aromatic hydrocarbons

Table S8. Baseline mean and 10th to 90th percentiles for creatinine-adjusted urine concentrations of B[a]P equivalents and Σ8 OH-PAHs. Baseline was measured before the three firefighting sessions; wood, gas and without fire. Baseline levels were measured during spring and autumn, where the spring baseline was used for comparison to the wood fire session and the autumn baseline was used for comparison to the gas fire session and the session without fire.

**Urine analysis** creatinine-adjusted	**Unit**	**Baseline Mean (10th to 90th percentiles)**	
		Wood baseline	Gas & Without fire baseline
Urine bioassay	ng B[a]P eq. /mol creatinine	0.84 (0.26-1.43)	0.84 (0.26-1.43)
Urine SPE-LC-MS/MS	µmol OH-PAH /mol creatinine	9.22 (2.43-14.89)	2.21 (1.25-3.90)

B[a]P eq, Benzo[a]pyrene equivalents; SPE-LC-MS/MS, solid phase extraction- liquid chromatography – tandem mass spectrometry; OH-PAH, hydroxylated polycyclic aromatic hydrocarbons

6. Levels per session for individual PAH and OH-PAH

Table S9. Median [IQR] levels of individual PAHs (in ng/cm^2) in skin wipes per measuring session analyzed by GC-MS/MS. Values are best estimates (<LOQ = best estimate).

	Baseline spring	Baseline autumn	Wood	Gas	Without fire
**Naphthalene**	0.01 [0, 0.06]	0 [0, 0]	0 [0, 0]	0 [0, 0.08]	0.05 [0, 0.10]
**Acenaphthylene**	0 [0, 0]	0.02 [0.01, 0.04]	0.03 [0, 0.04]	0 [0, 0.04]	0 [0, 0]
**Acenaphthene**	0.01 [0, 0.02]	0 [0, 0]	0 [0, 0]	0.01 [0, 0.3]	0 [0, 0]
**Fluorene**	0 [0, 0]	0 [0, 0]	0 [0, 0]	0 [0, 0]	0 [0, 0]
**Phenanthrene**	0.05 [0, 0.1]	0 [0, 0]	0.18 [0.11, 0.24]	0.05 [0.02, 017]	0 [0, 0.02]
**Anthracene**	0 [0, 0]	0 [0, 0]	0 [0, 0]	0 [0, 0]	0 [0, 0]
**Fluoranthene**	0 [0, 0]	0 [0, 0.03]	0.05 [0, 0.12]	0 [0, 0.02]	0 [0, 0]
**Pyrene**	0 [0, 0]	0 [0, 0]	0.08 [0, 0.09]	0 [0, 0.07]	0 [0, 0]
**Benz[a]anthracene**	0 [0, 0]	0 [0, 0]	0 [0, 0.02]	0 [0, 0]	0 [0, 0]
**Chrysene**	0 [0, 0]	0 [0, 0]	0 [0, 0]	0 [0, 0]	0 [0, 0]
**Benzo[b + k]fluoranthene**	0 [0, 0.19]	0 [0, 0]	0 [0, 0.01]	0 [0, 0]	0 [0, 0]
**Benzo[a]pyrene**	0 [0, 0]	0 [0, 0]	0 [0, 0]	0 [0, 0]	0 [0, 0]
**Dibenz[a,h]anthracene**	0 [0, 0]	0 [0, 0]	0 [0, 0]	0 [0, 0]	0 [0, 0]
**Ideno[1,2,3-cd]pyrene**	0 [0, 0.01]	0 [0, 0]	0 [0, 0.35]	0 [0, 0]	0 [0, 0]
**Benzo[g,h,i]perylene**	0 [0, 0]	0 [0, 0]	0 [0, 0]	0 [0, 0]	0 [0, 0]
**Sum PAH**	**0.24 [0.09, 0.45]**	**0.05 [0.04, 0.20]**	**0.57 [0.33, 0.71]**	**0.12 [0.08, 0.48]**	**0.09 [0.06, 0.15]**

IQR, inter-quartile range (25th and 75th percentiles); LOQ, limit of quantification; PAH, polycyclic aromatic hydrocarbons

Table S10. Median [IQR] levels of individual OH-PAHs in urine (in ng/mL adjusted for density) per measuring session analyzed by SPE-LC-MS/MS. Values are best estimates (<LOQ = best estimate).

	**Baseline spring**	**Baseline autumn**	**Wood**	**Gas**	**Without fire**
**1-OH-NAP**	0.4 [0.2, 0.9]	0.2 [0.2, 0.2]	0.7 [0.6, 1.0]	0.3 [0.2, 1.2]	0.4 [0.2, 0.5]
**2-OH-NAP**	17.5 [8.9, 27.6]	2.4 [1.8, 4.7]	11.6 [7.2, 21.1]	6.3 [4.8, 8.5]	4.3 [2.6, 10.5]
**2-OH-FLU**	0.2 [0.1, 0.3]	0.1 [0.1, 0.2]	0.2 [0.2, 0.3]	0.3 [0.2, 0.4]	0.1 [0.1, 0.1]
**2+3-OH-PHE**	0.2 [0.1, 0.4]	0.1 [0.1, 0.2]	0.2 [0.2, 0.3]	0.3 [0.2, 0.4]	0.1 [0.1, 0.2]
**1-OH-PHE**	0.2 [0.1, 0.3]	0.1 (0.1, 0.2]	0.2 (0.1, 0.2]	0.2 [0.2, 0.3]	0.1 [0.1, 0.2]
**4-OH-PHE**	0.0 [0.0, 0.0]	0.0 [0.0, 0.0]	0.0 [0.0, 0.0]	0.0 [0.0, 0.0]	0.0 [0.0, 0.0]
**1-OH-PYR**	0.1 [0.1, 0.2]	0.1 [0.0, 0.1]	0.2 [0.2, 0.2]	0.2 [0.1, 0.3]	0.1 [0.1, 0.2]
**Sum OH-PAH**	**18.5 [10.1, 29.5]**	**3.1 [2.5, 5.5]**	**13.2 [8.4, 23.9]**	**7.8 [6.3, 11.3]**	**4.9 [3.7, 12.4]**

1-OH-NAP, 1-hydroxynaphthalene; 2-OH-NAP, 2-hydroxynaphthalene; 2-OH-FLU, 2-hydroxyfluorene; 2+3-OH-PHE, 2- and 3-hydroxyphenanthrene; 1-OH-PHE, 1-hydroxyphenanthrene; 4-OH-PHE, 4-hydroxyphenanthrene; 1-OH-PYR, 1-hydroxypyrene; OH-PAH, hydroxylated polycyclic aromatic hydrocarbons.

7. Density-adjustment justification

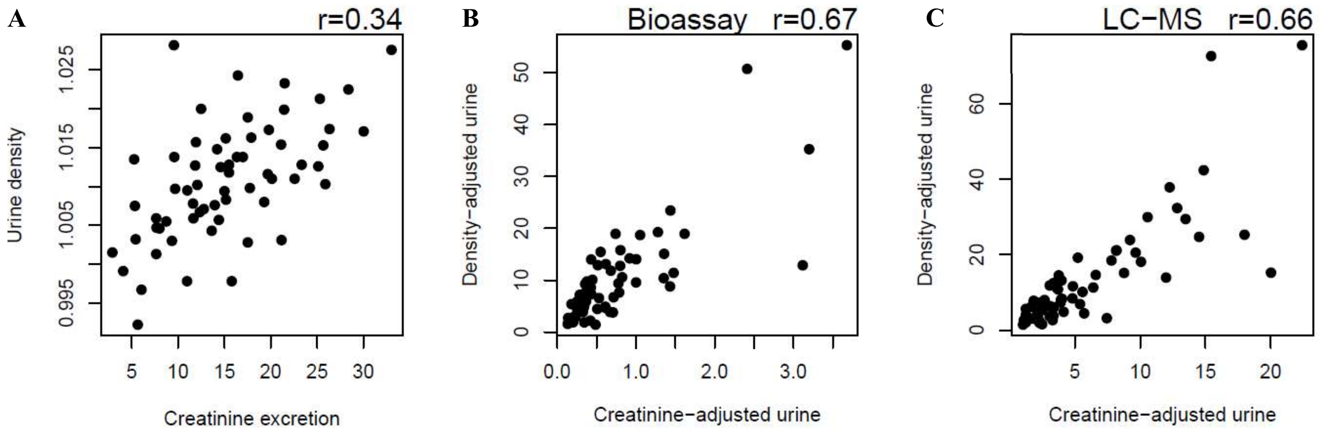

Figure S5. Linear regression analysis comparing A) urine density (g/mL) and creatinine excretion (mmol/L), and density-adjusted and creatinine-adjusted B) bioassay results (B[a]P equivalents) and C) SPE-LC-MS/MS results (OH-PAHs). The correlation coefficient *r* is indicated. Samples were collected from repeated measurements. Subjects (n) = 17.

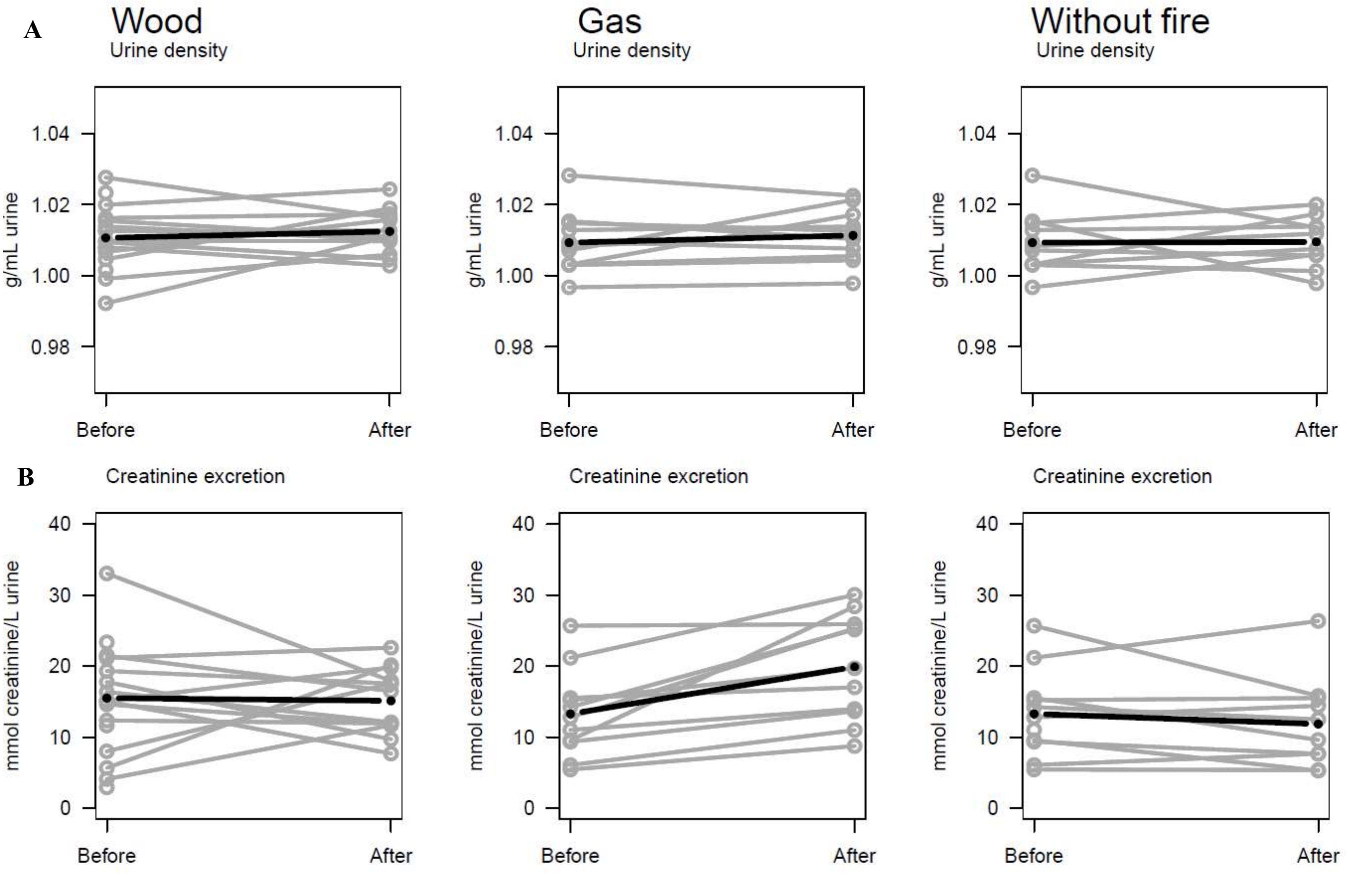

Figure S6. Difference in A) urine density (g/mL) and B) creatinine excretion (mmol/L) before and after the three firefighting sessions (wood, gas and without fire). Bold lines indicate the mixed effect linear regression. Samples were collected from repeated measurements where each point represents one measurement. Subjects (n = 14 (wood) & 11 (gas, without fire).

8. Levels of PAHs and B[a]P equivalents for creatinine-adjusted urine

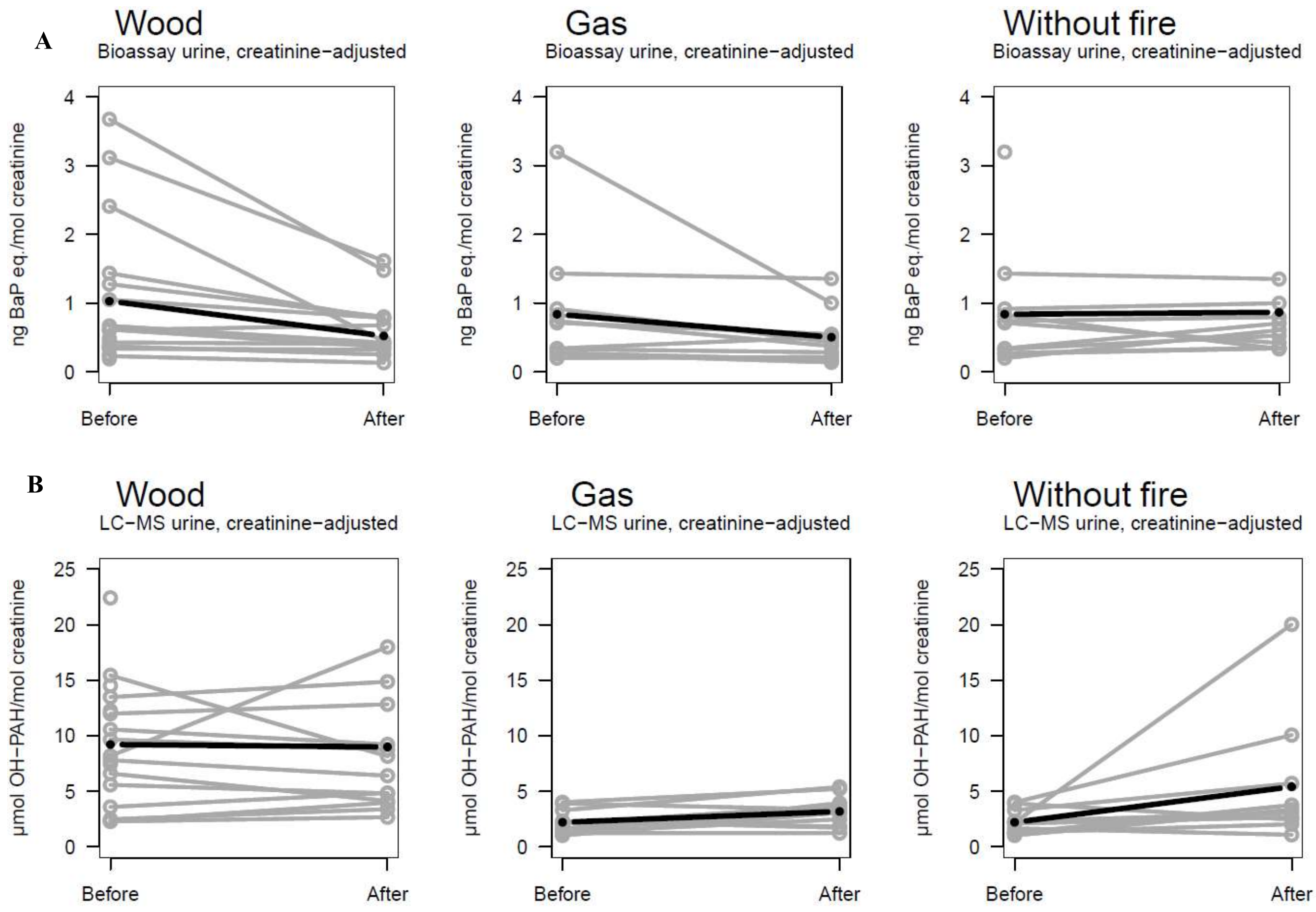

Figure S7. Difference in B[a]P equivalents (A) and Σ8 OH-PAHs (B) for creatinine-adjusted urine, measured by SPE-LC-MS/MS before and after the three firefighting sessions (wood, gas and without fire). The unit is µmol/mol creatinine. Bold lines indicate the mixed effect linear regression. Samples were collected from repeated measurements where each point represents one measurement. Subjects (n) = 14 (wood) & 11 (gas, without fire).

9. Effect sizes (percentage change) between baseline and after firefighting session for creatinine-adjusted urine

Table S11. Percentage change and confidence intervals (CI) per type of fire, indicating the difference between before and after the three firefighting sessions for creatinine-adjusted urine. Calculations are based on the LME model output. Bold text indicates significant values (p <0.05).

Analysis	Unit	Wood After vs before session		Gas After vs before session		Without fire After vs before session	
		% Change (95 % CI)	*p*-value	% Change (95 % CI)	*p*-value	% Change (95 % CI)	*p*-value
Urine bioassay	ng B[a]P eq./mol creatinine	**-33.5 (-54.4; -3.0)**	**0.034**	**-31.2 (-54.8; 4.8)**	**0.001**	8.6 (-29.5; 67.2)	0.71
Urine SPE- LC-MS/MS	µmol OH-PAH/mol creatinine	-2.3 (-26.3; 29.5)	0.87	**46.4 (7.0; 100.3)**	**0.017**	**86.1 (21.8; 184.2)**	**0.004**

10. Odds ratios for urine

Table S12. Odds ratios for density-adjusted urine results, obtained by logistic regression. Odds ratios were calculated using standard scores (Z-scores), derived from bioassay results, LC-MS results and bioassay and LC-MS results combined (mean).

Urine	Odds ratio	95 % CI
Urine bioassay	0.7	0.3; 1.2
Urine SPE-LC-MS/MS	0.9	0.5; 1.5
Urine Combined (bioassay & SPE-LC-MS/MS)	0.7	0.3; 1.4

11. Correlation bioassay and chemical analysis

Scatter plots were performed to investigate the linear regression between the bioassay data and the chemical analysis (GC-MS/MS and SPE-LC-MS/MS) data.

Urine

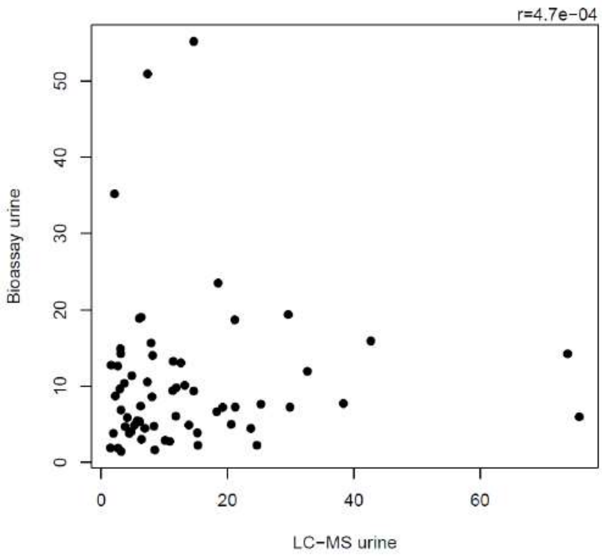

Figure S8. Linear regression analysis comparing urine bioassay results (B[a]P equivalents) and urine SPE-LC-MS/MS results (OH-PAHs). The correlation coefficient *r* is indicated. Samples were collected from repeated measurements. Subjects (n) = 17.

Skin wipes

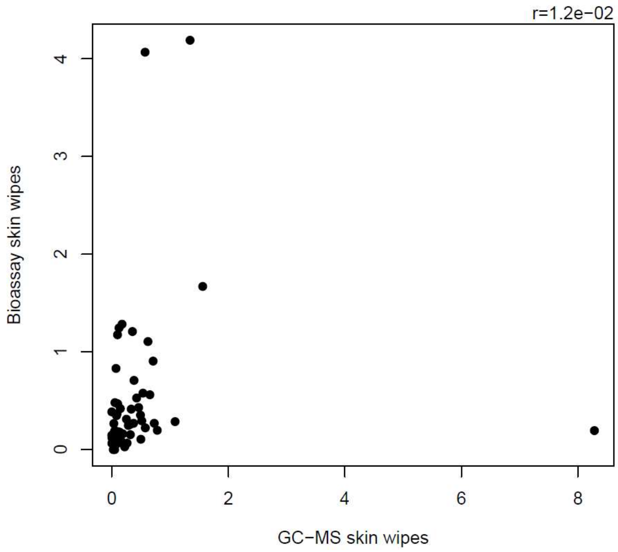

Figure S9. Linear regression analysis comparing skin bioassay results (B[a]P equivalents) and skin GC-MS/MS results (PAHs). The correlation coefficient *r* is indicated. Samples were collected from repeated measurements. Subjects (n) = 17.

References

1. Frederiksen, M., et al., *Online SPE-LC-MS-MS method for eight hydroxylated metabolites of polycyclic aromatic hydrocarbons in urine and determination of optimal sampling time after firefighter training.* Toxicology Letters, 2024. **400**: p. 9-15.
